# The role of a dairy fraction rich in milk fat globule membrane in the suppression of postprandial inflammatory markers and bone turnover in obese and overweight adults: an exploratory study

**DOI:** 10.1186/s12986-017-0189-z

**Published:** 2017-05-17

**Authors:** Tara S. Rogers, Elieke Demmer, Nancy Rivera, Erik R. Gertz, J. Bruce German, Jennifer T. Smilowitz, Angela M. Zivkovic, Marta D. Van Loan

**Affiliations:** 1 Department of Nutrition, University of California, Davis, 1 Shields Avenue, Davis, CA 95616 USA; 2 Center for Musculoskeletal Health, University of California, Davis Medical Center, 4625 2nd Avenue, Sacramento, CA 95817 USA; 3 USDA, Agricultural Research Service, Western Human Nutrition Research Center, 430 West Health Sciences Drive, Davis, CA 95616 USA; 4 Foods for Health Institute, University of California, Davis, 1 Shields Avenue, Davis, CA 95616 USA; 5 Department of Food Science & Technology, University of California, Davis, 1 Shields Avenue, Davis, CA 95616 USA

**Keywords:** Postprandial, Bone Turnover, Inflammation, Milk Fat Globule Membrane, C-telopeptide of type 1 collagen (CTX)

## Abstract

**Background:**

Inflammation is associated with increased bone resorption; the role of inflammation in postprandial bone turnover has not been explored. Consumption of milk fat globule membrane (MFGM) reduces inflammation in animal models. This study aimed to measure postprandial changes in bone turnover after intake of high saturated fat test meals, with- and without the anti-inflammatory ingredient MFGM.

**Methods:**

Subjects (*n* = 36 adults) were obese (BMI 30–39.9 kg/m^2^) or overweight (BMI 25–29.9 kg/m^2^) with two traits of Metabolic Syndrome. Subjects consumed a different test meal on four occasions at random; blood draws were taken at baseline and 1, 3, and 6 h postprandial. Test meals included whipping cream (WC), WC + MFGM, palm oil (PO) and PO + MFGM. Biomarkers of bone turnover and inflammation were analyzed from all four time points.

**Results:**

Test meal (treatment) by time interactions were significant for bone resorption marker C-telopeptide of type 1 collagen (CTX) (*p* < 0.0001) and inflammatory marker interleukin 10 (IL-10) (*p* = 0.012). Significant differences in overall postprandial response among test meals were found for CTX and soluble intercellular adhesion molecule (sICAM), with the greatest overall postprandial suppression of CTX occurring in meals containing MFGM. However, test meal by MFGM interactions were non- significant for bone and inflammatory markers. Correlations between CTX and inflammatory markers were non-significant.

**Conclusion:**

This exploratory analysis advances the study of postprandial suppression of bone turnover by demonstrating differing effects of high SFA meals that contained MFGM; however MFGM alone did not directly moderate the difference in postprandial CTX response among test meals in this analysis. These observations may be useful for identifying foods and ingredients which maximize the suppression of bone resorption, and for generating hypotheses to test in future studies examining the role of inflammation in postprandial bone turnover.

**Trial registration:**

Clinicaltrials.gov NCT01811329. Registered 11 March 2013.

**Electronic supplementary material:**

The online version of this article (doi:10.1186/s12986-017-0189-z) contains supplementary material, which is available to authorized users.

## Background

Biomarkers of bone resorption have been observed to fluctuate over the course of a day, with a peak occurring at night and a nadir seen in the day, particularly in the late afternoon. This pattern appears exaggerated with food intake [1–3]. Consumption of glucose suppresses bone resorption marker C- telopeptide of type I collagen (CTX) by 45–50% approximately 120 min after intake in healthy subjects [4, 5]. Mixed meals induce a similar but delayed reduction in CTX approximately 180 min after eating [5, 6]. In contrast to bone resorption, bone formation markers such as N-terminal serum type 1 procollagen (P1NP) change less drastically in the postprandial state [1, 4]. Explanations for the postprandial suppression of bone turnover have focused primarily on insulin and incretin hormones, but additional factors such as inflammatory mediators may contribute to the observed reduction in circulating CTX in the postprandial period [5].

In the Post-Prandial Inflammation (PPI) study, our group showed that the addition of a dairy fraction rich in milk fat globule membrane (MFGM) reduced postprandial concentrations of cholesterol, inflammatory markers and insulin in overweight and obese subjects who consumed test meals high in saturated fatty acids (SFA) [7]. MFGM was selected as a key ingredient due to its reported anti-inflammatory properties [8]. Composed of sphingolipids and glycerophospholipids, as well as proteins, MFGM covers the apical surface of lipid droplets produced by mammary glands [8, 9]. Whole buttermilk that was created during butter churning in decades past was a naturally rich source of MFGM, but today MFGM can be isolated, purified and added to other foods [9]. MFGM has been shown to reduce inflammation [10], improve endurance capacity and lipid metabolism [11] in animals, as well as reduce frailty in elderly women [12]. To date, effects of MFGM on bone outcomes have not been investigated in clinical studies. The PPI study provided the opportunity to conduct exploratory analyses of potential associations between postprandial bone turnover and postprandial inflammation using MFGM.

The aims of the present project were to examine postprandial changes in bone turnover after intake of high SFA challenge meals (with and without MFGM), and to investigate the relationships between the responses of inflammatory markers and bone turnover markers to the test meals. Since inflammation has been associated with increased bone resorption [13], and we previously showed that the addition of MFGM to a high SFA challenge meal reduces inflammatory markers [7], we hypothesized that due to anti-inflammatory effects of MFGM, high SFA challenge meals containing MFGM would attenuate postprandial bone turnover markers to a greater extent than high SFA challenge meals without MFGM.

## Methods

### Participants

Details of the PPI study have been previously published [7]. Briefly, subjects were recruited from the Davis and greater Sacramento areas of California and included 36 adults (19 women and 17 men). Inclusion criteria were 18–65 years of age and a body mass index (BMI) classified as obese (BMI 30–39.9 kg/m^2^) or overweight (BMI 25–29.9 kg/m^2^) plus two traits of Metabolic Syndrome (MetS). Per the American Heart Association definition, MetS traits include blood pressure ≥ 130/85 mmHg, fasting plasma triglyceride ≥ 150 mg/dl, fasting plasma high density lipoprotein (HDL) cholesterol < 40 mg/dl for men and < 50 mg/dl for women, waist circumference > 40 inches for men and 35 inches for women, and fasting glucose ≥ 100 mg/dl [14]. Exclusion criteria included gastrointestinal disorders, type 2 diabetes, immune-related disorders, cancer, self-reported eating disorder, use of anti-inflammatory pain medication, use of over the counter anti-obesity agents or corticosteroids in the last 12 weeks, initiation of statin therapy in the last 12 weeks, initiation of fish, krill, flax, borage or primrose seed oils within the last 12 weeks, use of dietary supplements with concentrated soy isoflavones, resveratrol or other polyphenols, initiation, change or cessation of hormonal birth control in the last 6 months, known allergy or intolerance to study food, adherence to a vegetarian diet, consumption of >1% of energy from trans-fats, > 1 serving of fish per week, > 14 grams of fiber per 1000 kcal/day, <16:1 total omega-6:omega 3 fatty acid ratio, >10% weight loss or gain in the past 6 months, poor vein assessment determined by phlebotomist, use of tobacco products, initiation of a new exercise program in the last month, and pregnancy, lactation, or plans to become pregnant in the next 6 months. Fulfillment of enrollment criteria was determined through questionnaires, analysis of a fasting blood sample for blood lipids and glucose, and anthropometric measurements (height, weight, waist circumference) that were taken during the subjects’ screening visits. The study protocol was approved by the Institutional Review Board of the University of California at Davis, and all procedures performed in the study were in accordance with the ethical standards of the 1964 Helsinki Declaration and its later amendments or comparable ethical standards. Informed consent was obtained from all individual participants included in the study. The study was registered at clinicaltrials.gov under NCT01811329.

### Study design

**Fig. 1:**
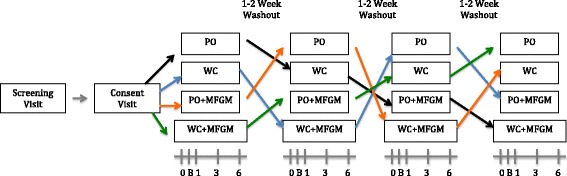
Study design. Test meals: palm oil (PO), palm oil plus milk fat globule membrane (PO + MFGM), whipping cream (WC), whipping cream plus milk fat globule membrane (WC + MFGM). Venipuncture timeline: 0 = baseline, B = breakfast test meal, 1 = 1 hour postprandial, 3 = 3 hours postprandial, 6 = 6 hours postprandial. *N* = 36

Phone screenings were used to determine subject eligibility, after which the individual reported to the Western Human Nutrition Research Center (WHNRC) to complete consent forms. Subjects were randomized to one of four treatment sequences in a repeated measures Latin Square design (Fig. 1). The advantage of this design for a repeated measures experiment is that it ensures a balanced fraction of all treatment combinations when subjects are limited and the sequence effect of treatment can be considered to be negligible. Investigators were blind to treatment order. A washout period of 1–2 weeks was observed between treatments to prevent carry-over effects across treatments. A random allocation sequence generator (randomization.com; seed#4234) was used to assign treatment order.

Subjects were instructed to abstain from alcohol, NSAIDs, and other anti-inflammatory supplements 72 h before each test day and from vigorous exercise and consuming seafood 24 h before each test day. Additionally, subjects recorded their diets 24 h before each test day. Nutrition Data System for Research (NDSR; University of Minnesota) was used to assess the 1-day diet record for compliance with the pre-study instructions.

The study took place at the WHNRC in Davis, CA. Subjects fasted for 10–12 h prior to each study day. Subjects completed a modified gastrointestinal questionnaire [15] and provided a fasting blood draw at the beginning of each study day. Blood pressure, heart rate, weight and waist circumference measurements were also recorded. Subjects then consumed the “breakfast” test meal within 20 min, and postprandial blood draws were taken at 1, 3, and 6 h (Fig. 1). Subjects were not allowed to consume any additional food throughout the study day but could drink bottled water ad libitum. Subjects were also instructed to minimize their physical activity during the remaining time of the test day by either staying at the test center for the entire 7-hour period or traveling by car if they chose to leave between blood draws. If subjects left and returned to the center, they were instructed to arrive 15 min prior to their scheduled blood draw to allow for a 10 min rest period before the venipuncture.

### Test meals

Meals consisted of a smoothie, as well as a bagel with strawberry preserves. Smoothies were made from whipping cream (WC) or palm oil (PO); milk fat globule membrane (MFGM) was added to one WC smoothie (WC + MFGM) and one PO smoothie (PO + MFGM). In this study, MFGM was sourced from the complex milk lipid fraction powder BPC50 (Fonterra Co-operative Group Ltd., Auckland, New Zealand) [16]. The composition of BPC50 includes the following (%wt/wt): 52% protein (13.2% membrane-derived protein), 6.6% lactose, and 36.2% total fat (22.5% triglycerides and 13.7% phospholipids, 0.63% gangliosides (GD3), and 5.2% ash) [17–19]. BPC50 contains the following MFGM-derived proteins in greatest abundance: fatty acid binding protein, butyrophilin, lactadherin, adipophilin, xanthine oxidase, and mucin [18]. Because the WC and PO smoothies did not contain BPC50, whey protein isolate was added to match the protein content. The nutrient composition of the test meals is shown in Additional file 1. Test meal ingredients are listed in the Additional file 2.

Each test meal was customized to provide 40% of each subject’s total energy intake (EI), as determined by the National Academy of Sciences equation from the Institute of Medicine Dietary Reference Intake. This equation accounted for gender, age, weight, height and physical activity [20]. The Baecke Physical Activity questionnaire was used to determine habitual physical activity [21]. The composition of each test meal was approximately 55% fat (49–87 grams per individual EI), 30% carbohydrate (61–107 grams per individual EI, and 15% protein (31–55 grams per individual EI). The NDSR was used to estimate the nutrient composition of each test meal. MFGM replaced 31% of the fat in each meal (34% of total kcal, 53.2–93.1) grams depending on individual EI). Per study protocol, subjects consumed each meal in its entirety, rinsed the beverage cup with bottled water and drank the rinse water.

### Blood analyses

A trained phlebotomist at the WHNRC collected blood by venipuncture at each time point. Whole blood was centrifuged in a tabletop ultracentrifuge for 15 min at 4 °C at 1300 × g within 30 min of collection. Plasma was then separated into 1.5 mL aliquots and immediately frozen at −70 °C until analysis. Serum was allowed to clot on ice for 30 min, centrifuged for 15 min at 4 °C at 1300 × g and transferred into 1.5 mL aliquots and frozen at −70 °C until analysis.

### Bone biomarkers

C-telopeptide of type 1 collagen (CTX) was measured by enzyme linked immune-sorbent assay (ELISA) (Immunodiagnostic Systems, Inc., Gaithersburg, MD, USA). Type 1 C-terminal collagen propeptide (C1CP) was also measured by ELISA (Quidel Corporation, San Diego, CA, USA).

### Inflammatory markers

Inflammatory biomarker analyses were conducted at all four time points. An electro-chemiluminescence detection system using multi-array technology (SECTOR Imager 2400, Meso Scale Discovery) was used to analyze interleukin-6 (IL-6), interleukin-18 (IL-18), interleukin-1 (IL-1), tumor necrosis factor alpha (TNFα), C-reactive protein (CRP) and soluble intercellular adhesion molecule (sICAM) per manufacturer’s instructions. Plasma was used to measure IL-18; serum was used to measure all other inflammatory markers. In brief, 25–50 μL of serum or plasma was added to pre-coated plates containing capture antibodies. After incubation, plates were washed, and a labeled detection antibody was added. The bound detection antibodies emit light upon electrochemical stimulation, and a plate reader was used to quantify each protein of interest.

### Metabolic parameters

Assessment of plasma glucose, triglycerides, HDL-cholesterol and insulin was completed at the clinical laboratory at the University of California Medical Center (Sacramento, CA) using standard clinical techniques.

### Clinical characteristics

Anthropometric data were collected at the time of screening. Measurements included height (Ayrton Stadiometer Model S100; Ayrton Corporation), body weight (6002 Wheelchair Scale; Scale-tronix), waist circumference measured in the standing position midway between the lower rib margin and ileac crest (QM2000 Measure Mate; QuickMedical), blood pressure and resting heart rate (Carescape V100 with Critikon Dura-cuff for either adults or large adults; GE Medical Instruments). BMI was calculated as kg/m^2^. Bone density was also measured by dual energy X-ray absorptiometry (DXA, Lunar Prodigy instrument; GE Medical Instruments).

### Statistical analyses

Sample size to detect a minimum significant difference between treatment groups was determined for the original PPI study, and power calculations were based on previously published plasma inflammatory marker data [22] and preliminary oxylipin studies in our lab. We defined the minimal detectable difference as the difference between the maximum and minimum responses; for example, the magnitude of this difference was 34.1 for prostaglandin E2 (PGE2). With a significance level set at a two-sided alpha of 0.05 and power held at 80%, we calculated that a sample size of 36 subjects was needed for this four-way crossover trial.

Descriptive statistics (mean ± standard deviation) were calculated for subjects’ baseline characteristics (Tables 1 and 2). Data were tested for normality with the Shapiro-Wilk test and transformed as appropriate. A repeated measures mixed model analysis (including the random effects of subject and subject by treatment) was used to test the effects of time, treatment (test meal) and time by treatment interaction for the bone marker variables (C1CP, CTX) and inflammatory variables (IL-6, IL-18, TNF-α, CRP, IL-10, sICAM) (Table 3).

**Table 1:** Subject baseline characteristics^a^

	Mean ± SD	MetS criteria^b^
Age (years)	42.9 ± 14.0	---
Weight (kg)	92.9 ± 12.2	---
Height (m)	1.7 ± 0.1	---
BMI, (kg/m^2^)	31.7 ± 2.6	---
Total Body Fat (%)	36.7 ± 7.8	---
Total Body Fat, Male (%)	30.9 ± 6.2	---
Total Body Fat, Female (%)	41.9 ± 4.9	---
Waist circumference (inches)	39.3 ± 3.2	---
Waist circumference, Male (inches)^c^	41.1 ± 3.1	>40
Waist circumference, Female (inches)^c^	37.9 ± 2.2	>35
Systolic blood pressure (mmHg)	123.9 ± 13.6	≥130
Diastolic blood pressure (mmHg)	75.0 ± 10.3	≥85
HDL cholesterol (mg/dl)	48.3 ± 14.1	---
HDL cholesterol, Male (mg/dl)^c^	43.2 ± 11.7	<40
HDL cholesterol, Female (mg/dl)^c^	52.9 ± 14.7	<50
Fasting glucose (mg/dl)	91.0 ± 7.4	≥100
Fasting triglycerides (mg/dl)	122.5 ± 57.8	≥150

^a^Measurements taken at screening visit (*n* = 36). *BMI* body mass index, *HDL* high-density lipoprotein

^b^MetS as defined by the American Heart Association [14]. *MetS* metabolic syndrome

^c^Male *n* = 17, Female *n* = 19

**Table 2:** Bone mineral measurements^a^ of subjects at baseline (Mean ± SD)

	BMC (g)	BMD (g/cm^2^)	T-score
Males (*n* = 17)			
Lumbar Spine L1-L4	81.00 ± 13.3	1.13 ± 0.1	0.39 ± 1.1
Total Right Hip	48.83 ± 8.7	1.12 ± 0.1	0.58 ± 0.9
Total Left Hip	46.81 ± 14.0	1.12 ± 0.1	0.61 ± 0.9
Females (*n* = 19)			
Lumbar Spine L1-L4	62.17 ± 8.6	1.05 ± 0.1	0.05 ± 1.0
Total Right Hip	31.49 ± 8.2	0.98 ± 0.1	0.33 ± 1.0
Total Left Hip	34.29 ± 5.0	1.00 ± 0.1	0.43 ± 1.0

^a^Measurements taken by dual energy X-ray absorptiometry, *BMC* bone mineral content, *BMD* bone mineral density, T score = comparison to young adult average

**Table 3:** Concentrations of postprandial bone biomarkers and inflammatory markers (Mean ± SD)

	Postprandial time point						
	0 hour	1 hour	3 hour	6 hour	Treatment effect *p*-value	Time effect *p*-value	Time x treatment interaction *p*-value
C1CP (ng/ml)					0.093	0.084	0.195
PO + MFGM	134.4 ± 140.6	131.1 ± 134.1	128.1 ± 119.9	131.3 ± 127.7			
PO	122.0 ± 133.7	127.1 ± 138.7	117.5 ± 124.2	127.9 ± 152.0			
WC + MFGM	130.0 ± 135.2	126.2 ± 137.0	130.1 ± 146.5	129.3 ± 125.9			
WC	153.1 ± 269.9	137.8 ± 201.7	123.6 ± 133.8	129.2 ± 146.3			
CTX (ng/ml)					0.0001	<0.0001	<.0001
PO + MFGM	0.57 ± 0.27	0.33 ± 0.14	0.24 ± 0.12	0.32 ± 0.16			
PO	0.53 ± 0.22	0.31 ± 0.13	0.24 ± 0.12	0.44 ± 0.22			
WC + MFGM	0.56 ± 0.27	0.33 ± 0.16	0.22 ± 0.11	0.26 ± 0.14			
WC	0.55 ± 0.26	0.31 ± 0.11	0.20 ± 0.09	0.38 ± 0.20			
IL-6 (pg/ml)					0.449	<0.0001	0.975
PO + MFGM	0.72 ± 1.37	0.59 ± 1.26	0.54 ± 0.98	0.76 ± 1.40			
PO	0.74 ± 1.08	0.59 ± 0.87	0.61 ± 1.09	0.76 ± 1.16			
WC + MFGM	0.78 ± 1.43	0.63 ± 1.27	0.60 ± 1.20	0.86 ± 1.48			
WC	0.70 ± 1.10	0.63 ± 1.18	0.60 ± 1.13	0.87 ± 1.51			
IL-18 (pg/ml)					0.671	<0.0001	0.245
PO + MFGM	11.11 ± 3.33	11.32 ± 3.32	10.72 ± 2.74	10.80 ± 3.39			
PO	11.24 ± 3.10	10.51 ± 3.34	9.629 ± 3.15	10.96 ± 2.95			
WC + MFGM	11.79 ± 3.64	10.63 ± 4.11	10.40 ± 3.67	10.81 ± 3.17			
WC	11.05 ± 3.44	10.98 ± 3.03	10.09 ± 2.69	10.18 ± 3.06			
TNFα (pg/ml)					0.679	<0.0001	0.303
PO + MFGM	2.34 ± 0.67	2.36 ± 0.66	2.27 ± 0.62	2.22 ± 0.68			
PO	2.49 ± 0.71	2.36 ± 0.56	2.29 ± 0.63	2.36 ± 0.64			
WC + MFGM	2.54 ± 0.83	2.36 ± 0.81	2.32 ± 0.81	2.32 ± 0.73			
WC	2.44 ± 0.70	2.38 ± 0.67	2.27 ± 0.57	2.32 ± 0.63			
CRP (mg/l)					0.207	<0.0001	0.608
PO + MFGM	1.49 ± 0.85	1.51 ± 0.86	1.46 ± 0.85	1.52 ± 0.87			
PO	1.55 ± 0.86	1.65 ± 0.87	1.58 ± 0.88	1.62 ± 0.87			
WC + MFGM	1.49 ± 0.85	1.56 ± 0.90	1.51 ± 0.87	1.56 ± 0.88			
WC	1.49 ± 0.83	1.58 ± 0.90	1.50 ± 0.84	1.53 ± 0.86			
IL-10 (pg/ml)					0.278	0.206	0.012
PO + MFGM	0.49 ± 1.17	0.52 ± 1.19	0.52 ± 1.12	0.57 ± 1.14			
PO	0.56 ± 1.37	0.54 ± 1.35	0.54 ± 1.22	0.51 ± 1.32			
WC + MFGM	0.52 ± 1.08	0.57 ± 1.09	0.57 ± 1.04	0.58 ± 1.17			
WC	0.52 ± 1.13	0.58 ± 1.15	0.58 ± 1.21	0.55 ± 1.28			
sICAM (mg/l)					0.168	<0.0001	0.114
PO + MFGM	0.96 ± 0.51	0.97 ± 0.51	0.95 ± 0.51	0.97 ± 0.53			
PO	0.97 ± 0.51	1.058 ± 0.54	1.00 ± 0.51	1.044 ± 0.54			
WC + MFGM	0.93 ± 0.50	0.99 ± 0.53	0.97 ± 0.54	0.97 ± 0.51			
WC	0.92 ± 0.48	1.00 ± 0.52	0.96 ± 0.49	0.96 ± 0.49			

Actual (untransformed) values are presented here; analysis was done on the transformed data. *N* = 36

Test meals: palm oil plus milk fat globule membrane (PO + MFGM), palm oil (PO), whipping cream plus milk fat globule membrane (WC + MFGM), whipping cream (WC)

Bone variables: type 1 C-terminal collagen propeptide (C1CP), C-telopeptide of type 1 collagen (CTX)

Inflammatory variables: interleukin-6 (IL-6), interleukin-18 (IL-18), tumor necrosis factor alpha (TNFα), C-reactive protein (CRP), interleukin-10 (IL10), soluble intercellular adhesion molecule (sICAM)

Additionally, a summary measure (incremental area under the curve, iAUC) using the trapezoid method, was used for comparison of overall postprandial responses among test meals [23]. For bone markers and inflammatory variables, the mean incremental area under the transformed curve (iAUtC) of each test meal was compared by repeated measures analysis of covariance, including fixed effects of group and subject, and transformed hour 0 as a covariate (Table 4).

**Table 4:** Postprandial response (iAUtC^1^ ± SD) of bone biomarkers and inflammatory markers by test meal

	Test meals				Test Meal x MFGM interaction *p*-value
	PO + MFGM	PO	WC + MFGM	WC	
C1CP	−0.18 ± 0.9	0.07 ± 1.2	0.01 ± 0.9	−0.28 ± 0.8	0.859
CTX	−1.52 ± 1.0^b^	−1.16 ± 0.9^a^	−1.67 ± 0.9^c^	−1.49 ± 0.9^bc^	0.332
IL-6	−0.70 ± 1.5	−0.78 ± 1.4	−0.84 ± 1.5	−0.47 ± 1.0	0.846
IL-18	−1.93 ± 10.6	−5.58 ± 11.4	−6.68 ± 11.0	−3.81 ± 11.5	0.106
TNF-α	−0.12 ± 0.7	−0.36 ± 0.6	−0.49 ± 0.7	−0.26 ± 0.7	0.102
CRP	0.04 ± 0.3	0.12 ± 0.2	0.10 ± 0.2	0.08 ± 0.2	0.119
IL-10	0.71 ± 1.8	−0.25 ± 1.8	0.37 ± 1.7	0.19 ± 1.7	0.567
sICAM	0.13 ± 0.5^b^	0.37 ± 0.4^a^	0.28 ± 0.4^ab^	0.32 ± 0.5^ab^	0.080

^1^Mean incremental area under transformed curve

Significant differences in iAUtC of bone biomarkers and inflammatory markers among test meals are indicated by superscript letters

*N* = 36

Test meals: palm oil plus milk fat globule membrane (PO + MFGM), palm oil (PO), whipping cream plus milk fat globule membrane (WC + MFGM), whipping cream (WC)

Bone variables: type 1 C-terminal collagen propeptide (C1CP), C-telopeptide of type 1 collagen (CTX)

Inflammatory variables: interleukin-6 (IL-6), interleukin-18 (IL-18), tumor necrosis factor alpha (TNFα), C-reactive protein (CRP), interleukin-10 (IL-10), soluble intercellular adhesion molecule (sICAM)

To examine whether postprandial changes in inflammatory variables may have mediated postprandial changes in the bone markers, within-subject correlations between iAUtC of inflammatory variables and iAUtC of bone markers were examined, controlling for hour 0 values (Table 5). Correlation coefficients were calculated as partial Pearson correlations; mixed model regression (with a random effect of subject) was used to calculate p-values for each correlation in order to account for the fact that the four measurements were not independent of each other. Additionally, we calculated correlations between CTX and selected metabolic variables (glucose, HDL cholesterol, triglycerides and insulin) at each time point (Additional file 3).

**Table 5:** Within-subject correlations of bone biomarkers and inflammatory markers^a^

Inflammatory variable	C1CP correlation coefficient	*p-*value	CTX correlation coefficient	*p*-value
IL-6	−0.20	0.045	−0.08	0.421
IL-18	−0.09	0.338	−0.12	0.202
TNF-α	−0.15	0.118	0.05	0.613
CRP	0.14	0.159	0.08	0.445
IL-10	0.02	0.877	−0.10	0.307
sICAM	0.03	0.748	0.01	0.935

^a^Correlations calculated for incremental area under the transformed curve, controlling for hour 0 values of the two variables. Correlation coefficients were calculated as partial Pearson correlations; mixed model regression (with a random effect of subject) was used to calculate p-values for each correlation in order to account for the fact that the four measurements were not independent of each other. *N* = 36

Bone variables: type 1 C-terminal collagen propeptide (C1CP), C-telopeptide of type 1 collagen (CTX)

Inflammatory variables: interleukin-6 (IL-6), interleukin-18 (IL-18), tumor necrosis factor alpha (TNFα), C-reactive protein (CRP), interleukin-10 (IL-10), soluble intercellular adhesion molecule (sICAM)

GraphPad Prism 6.0c (GraphPad Software, Inc., La Jolla, CA) and SAS for Windows release 9.4 (SAS Institute, Cary, NC) were used for statistical analyses.

## Results

Out of 207 potential subjects who were screened, 38 subjects were enrolled. Two subjects were disqualified due to initiation of medication that could confound the results and to scheduling difficulties. The final subject population included 17 males and 19 females. Details of subject enrollment, CONSORT Diagram and follow up have been previously published [7]. All subjects (*n* = 36) consumed all four test meals. One subject did not complete the postprandial blood draws after the PO + MFGM test meal due to difficulties with the venipuncture. The missing data for the 1, 3 and 6 h time points for this subject were accounted for in the statistical analyses per SAS protocol. For the correlations, the missing points were omitted from the analysis. For the various mixed model analyses, the non-missing points were included, and the missing points were excluded, but the nature of the model is that it implicitly imputes the missing values when estimating differences between the means for the treatments or the time points.

Baseline characteristics of the subjects were assessed at the consent visit and are shown in Table 1. Subjects were predominately Caucasian (67%) or Hispanic (28%). On average, subjects were obese and met MetS criteria for waist circumference and HDL cholesterol. Mean bone mineral content (BMC), bone mineral density (BMD), and T scores of male and female subjects are shown in Table 2. Bone measurements were taken once during the study to establish the baseline bone health of each subject. Mean T-scores for lumbar spine, total right hip and total left hip were within the normal range for male and female subjects.

Effects of treatment and time were significant for CTX (p ≤ 0.0001), and significant effects of time (*p* < 0.0001) were also observed for IL-6, IL-18, TNF-α, CRP and sICAM (Table 3). There were significant treatment by time interactions for CTX (*p* < 0.0001) and IL-10 (*p* = 0.012) (Table 3).

**Fig. 2:**
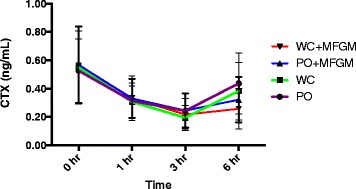
Postprandial CTX concentrations over time after consumption of four high SFA test meals. Test meals: palm oil (PO), palm oil plus milk fat globule membrane (PO + MFGM), whipping cream (WC), whipping cream plus milk fat globule membrane (WC + MFGM). *N* = 36

Postprandial CTX concentrations (mean ± SD) after each test meal are presented in Fig. 2. Differences in CTX response to the test meals were most apparent at 6 h postprandial; CTX concentrations remained lower at this time point after consumption of WC + MFGM and PO + MFGM test meals compared to WC and PO test meals.

Significant differences in overall postprandial response (iAUtC) among the test meals were found for CTX and sICAM. Test meal by MFGM interactions were not significant for any of the bone or inflammatory markers (Table 4).

The correlation between the iAUtCs of C1CP and IL-6 was statistically significant (*r* = −0.20; *p* = 0.045), but other correlations between bone markers and inflammatory variables were non-significant (Table 5).

## Discussion

This exploratory study is the first to examine the potential role of inflammation in postprandial bone turnover. In line with previous reports of postprandial bone turnover [5, 6] and as we observed with some of the inflammatory markers [7], bone resorption marker CTX significantly changed over time in the postprandial state. Interestingly, we observed that meals containing MFGM induced the greatest overall suppression of CTX, particularly at 6 h postprandial; however, test meal by MFGM interactions were non-significant. Furthermore, although a weak correlation was found between bone formation marker C1CP and IL-6, correlations between CTX and the inflammatory variables were not statistically significant. Taken together, these data suggest that suppression of postprandial inflammation may have a minimal role in the suppression of postprandial bone turnover and that MFGM did not directly moderate the difference in postprandial CTX response among test meals. However, further studies are warranted to confirm these observations.

The mechanisms of postprandial suppression of bone turnover are incompletely understood. During fasting (and especially during overnight fasts) the body must mobilize nutrients from the bone for calcium homeostasis and cell growth processes, and therefore bone resorption increases. It has been hypothesized that since organic compounds and nutrients are readily available to the skeleton after eating, bone resorption is unnecessary in the postprandial state [1]. Differing effects of nutrients and foods on postprandial bone resorption have been reported. A small study of healthy adults compared carbohydrate, fat and protein and found that all macronutrients suppressed postprandial CTX compared to baseline levels; however the postprandial CTX response to fat ingestion was more blunted [24]. In contrast, other researchers have observed a trend for greater CTX suppression with lower protein, higher fat foods [2]. The present study demonstrates differences in postprandial CTX suppression among high SFA meals containing an ingredient with anti-inflammatory properties (MFGM).

Previous work has revealed that bile acids [25], insulin [5] and incretin hormones such as glucose dependent insulinotropic peptide (GIP) may contribute to postprandial suppression of bone resorption [24, 26]; however, the PPI protocol did not include any measurements of bile acids or incretin hormones, or insulin at 6 h postprandial. Per the PPI protocol, insulin was measured at 0, 1 and 3 h postprandial, and we examined correlations between CTX and other selected metabolic variables (glucose, HDL cholesterol, triglycerides and insulin) at each time point separately. Other studies have suggested associations between bone turnover and cholesterol [27, 28], so the positive correlations that we observed between CTX and HDL cholesterol and negative correlations between CTX and triglycerides warrant further study (Additional file 3). We found no significant correlations between CTX and insulin at any of the three time points (Additional file 3).

We may have been unable to detect significant test meal by MFGM interactions due to the fact that the test meals were not matched for individual SFA’s (Additional file 1). Additionally, the PPI nutrient analysis did not include micronutrients such as calcium, which may influence bone turnover in the postprandial state [29]. Moreover, the study was designed to compare effects of the test meals against each other, so there was no control group with which to make comparisons. Furthermore, the sample size was calculated for the original PPI study based on plasma inflammatory marker and oxylipin data rather than bone turnover markers. Our sample size (*n* = 36) is comparable to that of other crossover studies using bone turnover markers [5, 30]; it is unlikely that the sample size was a major limitation in these exploratory analyses but this remains a possibility. Lastly, our protocol allowed subjects to leave the WHNRC if they traveled by car and returned 15 min early and rested for 10 min prior to their scheduled blood draw. In using this particular protocol, we intended to balance the need to minimize exercise for the sake of the experiment with the need to accommodate the free-living subjects who volunteered for our study. A total of 27 subjects chose to leave between blood draws, and it is possible that their activities while away from the WHNRC could have influenced our results. Future studies should follow a more stringent protocol that does not allow subjects to leave between blood draws.

Despite the aforementioned limitations, our observations may be useful for identifying foods and ingredients which maximize the suppression of bone resorption for therapeutic purposes, and for generating hypotheses to test in future studies. Identifying foods and ingredients (e.g. MFGM), as well as meal patterns (e.g. six small meals vs. three larger meals), which can suppress bone resorption for a longer period in the postprandial state, may lead to osteoporosis prevention strategies in the long term. Additionally, it is possible that MFGM may have interacted with other ingredients or nutrients in the meal to give the observed response. The possible interactive effects of MFGM with other food ingredients, as well as the effects of MFGM on longer-term bone outcomes such as BMD, will require further investigation.

## Conclusions

In this exploratory study, we have shown that bone resorption (as measured by the biomarker CTX) is significantly suppressed in the postprandial state, particularly after consumption of high SFA meals that contain MFGM. However, MFGM was not found to be a direct moderator of the CTX response. To further elucidate the potential role of inflammation in postprandial bone turnover, future studies based on our observations should 1) match test meals for individual fatty acids and micronutrients as well as macronutrients, 2) include measurements of postprandial bile acids, incretin hormones and insulin, and 3) assess interactions of MFGM with other food ingredients.

## Additional files

Additional file 1: Nutrient composition of test meals. (DOCX 21 kb)

Additional file 2: Test meal ingredient list. (DOCX 14 kb)

Additional file 3: Correlations of CTX and selected metabolic variables after intake of high saturated fat test meals. (DOCX 17 kb)

## Abbreviations

MFGM: Milk fat globule membrane
PO: Palm oil
PPI: Postprandial inflammation
WC: Whipping cream
WHNRC: Western Human Nutrition Research Center
