# Supplementary material for: The role of a dairy fraction rich in milk fat globule membrane in the suppression of postprandial inflammatory markers and bone turnover in obese and overweight adults: an exploratory study
Source: Nutr Metab (Lond). 2017 May 17;14:36. doi: 10.1186/s12986-017-0189-z (PMC5436451; doi:10.1186/s12986-017-0189-z)
Supplement: Supplementary file 1 — Nutrient composition of test meals. (DOCX 21 kb) [file 12986_2017_189_MOESM1_ESM.docx]

Additional file 1. Nutrient composition of test meals^1^

a) Palm oil-based test meals

|  | PO | PO+MFGM |
| --- | --- | --- |
| Energy (kcal) | 1086 ±187 | 1086 ± 186 |
| Protein (g) | 42.4 ±7.3 | 42.9 ± 7.4 |
| Fat (g) | 67.1 ±11.5 | 67.3 ± 11.6 |
| Carbohydrate (g) | 82.6 ±14.2 | 83.1 ± 14.3 |
| SFA 4:0 butyric acid (%)* | 0.01 ± 0.00 | 0.18 ± 0.03 |
| SFA 6:0 caproic acid (%)* | 0.00 ± 0.00 | 0.26 ± 0.04 |
| SFA 8:0 caprylic acid (%)* | 0.10 ± 0.02 | 0.24 ± 0.04 |
| SFA 10:0 capric acid (%)* | 0.02 ± 0.00 | 0.48 ± 0.08 |
| SFA 12:0 lauric acid (%)* | 0.16 ±0.03 | 0.88 ± 0.15 |
| SFA 14:0 myristic acid (%)* | 0.70 ± 0.12 | 2.52 ± 0.43 |
| SFA 16:0 palmitic acid (%)* | 29.53 ± 5.08 | 25.32 ± 4.35 |
| SFA 18:0 stearic acid (%)* | 2.67 ± 0.46 | 4.58 ± 0.79 |
| MUFA 16:1 palmitoleic acid (%)* | 0.12 ± 0.02 | 0.38 ± 0.06 |
| MUFA 18:1 oleic acid* | 26.67 ± 4.58 | 23.78 ± 4.08 |
| PUFA 18:2 linoleic acid* | 6.50 ± 1.12 | 5.03 ± 0.86 |
| PUFA 18:3 linolenic acid* | 0.30 ± 0.05 | - 1. ± 0.09 |

b) Whipping cream-based test meals

|  | WC | WC+MFGM |
| --- | --- | --- |
| Energy (kcal) | 1092 ± 187 | 1092 ± 188 |
| Protein (g) | 42.8 ± 7.3 | 43.2 ± 7.4 |
| Fat (g) | 67.8 ± 11.6 | 67.8 ± 11.7 |
| Carbohydrate (g) | 83.5 ± 14.3 | 83.9 ± 14.4 |
| SFA 4:0 butyric acid (%)* | 2.15 ± 0.37 | 1.67 ± 0.29 |
| SFA 6:0 caproic acid (%)* | 1.27 ± 0.22 | 1.08 ± 0.19 |
| SFA 8:0 caprylic acid (%) | 0.74 ± 0.13 | 0.68 ± 0.12 |
| SFA 10:0 capric acid (%) | 1.66 ± 0.29 | 1.59 ± 0.27 |
| SFA 12:0 lauric acid (%) | 1.86 ± 0.32 | 2.01 ± 0.35 |
| SFA 14:0 myristic acid (%) | 6.67 ± 1.15 | 6.54 ± 1.12 |
| SFA 16:0 palmitic acid (%) | 17.56 ± 3.01 | 17.31 ± 2.97 |
| SFA 18:0 stearic acid (%) | 8.07 ± 1.39 | 8.19 ± 1.41 |
| MUFA 16:1 palmitoleic acid (%)* | 1.50 ± 0.26 | 1.32 ± 0.23 |
| MUFA 18:1 oleic acid | 16.89 ± 2.90 | 17.19 ± 2.96 |
| PUFA 18:2 linoleic acid | 2.09 ± 0.36 | 2.09 ± 0.36 |
| PUFA 18:3 linolenic acid | 1.12 ± 0.19 | 1.06 ± 0.18 |

^1^ PO (palm oil); PO+MFGM (palm oil plus milk fat globule membrane); WC (whipping cream), WC+MFGM (whipping cream plus milk fat globule membrane). MFGM replaced 31% of the fat in each meal (34% of total kcal, 53.2-93.1) grams depending on individual estimated total energy intake). Data reported as mean ± standard deviation for all test meals (n=36). Test meal composition compared by Mann-Whitney test, and significant differences (p<0.05) are noted (*).
