# Supplementary material for: The role of a dairy fraction rich in milk fat globule membrane in the suppression of postprandial inflammatory markers and bone turnover in obese and overweight adults: an exploratory study
Source: Nutr Metab (Lond). 2017 May 17;14:36. doi: 10.1186/s12986-017-0189-z (PMC5436451; doi:10.1186/s12986-017-0189-z)
Supplement: Supplementary file 2 — Test meal ingredient list. (DOCX 14 kb) [file 12986_2017_189_MOESM2_ESM.docx]

Additional file 2. Test meal ingredient list^1^

|  | PO | PO+MFGM | WC | WC+MFGM |
| --- | --- | --- | --- | --- |
| Plain Bagel Thins (Thomas, Bimbo Bakeries, Horsham, PA, USA) | X | X | X | X |
| Strawberry Preserves (Safeway Select, Pleasanton, CA, USA) | X | X | X | X |
| Smoothie | | | | |
| DI Water (Davis, CA, USA) | X | X | X | X |
| Whey Protein Isolate (BioChem, Country Life, Hauppauge, NY, USA) | X |  | X |  |
| Cream of Tartar (Safeway Select, Pleasanton, CA, USA) | X | X |  |  |
| Palm Oil Shortening (Spectrum, Boulder, CO, USA) | X | X |  |  |
| BPC50 (Fontarra, Auckland, New Zealand) |  | X |  | X |
| Raspberry Sorbet (Cioa Bella, Florham Park, NJ, USA) | X | X | X | X |
| Heavy Whipping Cream (Lucerne, Pleasanton, CA) |  |  | X | X |

^1^ PO (palm oil); PO+MFGM (palm oil plus milk fat globule membrane); WC (whipping cream), WC+MFGM (whipping cream plus milk fat globule membrane)
