# Supplementary material for: The role of a dairy fraction rich in milk fat globule membrane in the suppression of postprandial inflammatory markers and bone turnover in obese and overweight adults: an exploratory study
Source: Nutr Metab (Lond). 2017 May 17;14:36. doi: 10.1186/s12986-017-0189-z (PMC5436451; doi:10.1186/s12986-017-0189-z)
Supplement: Supplementary file 3 — Correlations of CTX and selected metabolic variables after intake of high saturated fat test meals. (DOCX 17 kb) [file 12986_2017_189_MOESM3_ESM.docx]

Additional file 3. Correlations of CTX^1^ and selected metabolic variables after intake of high saturated fat test meals

| Test meal^2^ | Time point (hours postprandial) | Glucose (mg/dl) | HDL cholesterol (mg/dl) | Triglycerides (mg/dl) | Insulin^3^ (µIU/ml) |
| --- | --- | --- | --- | --- | --- |
| PO | 0 | -0.37 (0.030) | 0.15 (0.385) | -0.30 (0.081) | -0.08 (0.650) |
|  | 1 | -0.13 (0.454) | 0.14 (0.418) | -0.25 (0.145) | -0.03 (0.876) |
|  | 3 | -0.08 (0.641) | 0.15 (0.390) | -0.33 (0.055) | -0.15 (0.400) |
|  | 6 | -0.06 (0.714) | 0.27 (0.116) | -0.19 (0.266) | --- |
| WC | 0 | -0.22 (0.194) | 0.35 (0.042) | -0.34 (0.043) | -0.09 (0.600) |
|  | 1 | 0.10 (0.551) | 0.34 (0.049) | -0.51 (0.002) | -0.03 (0.883) |
|  | 3 | 0.06 (0.723) | 0.43 (0.010) | -0.37 (0.029) | -0.13 (0.468) |
|  | 6 | -0.05 (0.773) | 0.32 (0.058) | -0.35 (0.042) | --- |
| PO+MFGM | 0 | -0.33 (0.055) | 0.36 (0.034) | -0.23 (0.178) | -0.08 (0.641) |
|  | 1 | -0.15 (0.397) | 0.24 (0.159) | -0.30 (0.082) | -0.25 (0.148) |
|  | 3 | -0.12 (0.499) | 0.19 (0.273) | -0.33 (0.054) | -0.13 (0.475) |
|  | 6 | 0.04 (0.835) | 0.20 (0.249) | -0.11 (0.545) | --- |
| WC+MFGM | 0 | -0.18 (0.292) | 0.23 (0.184) | -0.45 (0.007) | -0.26 (0.128) |
|  | 1 | 0.35 (0.040) | 0.23 (0.194) | -0.48 (0.003) | 0.02 (0.902) |
|  | 3 | -0.08 (0.654) | 0.33 (0.055) | -0.42 (0.012) | -0.19 (0.284) |
|  | 6 | 0.16 (0.374) | 0.44 (0.008) | -0.29 (0.089) | --- |

Correlation coefficients (p-values) are presented. N=36

^1^ CTX (C-telopeptide of type 1 collagen; ng/mL)

^2^ PO (palm oil); PO+MFGM (palm oil plus milk fat globule membrane); WC (whipping cream), WC+MFGM (whipping cream plus milk fat globule membrane).

^3^Measured at 0 hours, 1 hour and 3 hours postprandial
